# Editorial: Role of mitochondria-associated non-coding RNAs in intracellular communication

**DOI:** 10.3389/fphys.2022.980674

**Published:** 2022-08-22

**Authors:** Veronica A. Burzio, Eric Barrey, Eleonora Leucci, Nina Entelis, John M. Hollander, Samarjit Das

**Affiliations:** ^1^ Centro Ciencia and Vida, Andes Biotechnologies SpA, Universidad Andrés Bello, Santiago, Chile; ^2^ Université Paris-Saclay, AgroParisTech, INRAE, GABI, Jouy-en-Josas, France; ^3^ Laboratory for RNA Cancer Biology, Department of Oncology, Katholieke Universiteit Leuven, Leuven, Belgium; ^4^ UMR 7156 GMGM Strasbourg University–CNRS, Strasbourg, France; ^5^ Division of Exercise Physiology, and Mitochondria, Metabolism & Bioenergetics Working Group, West Virginia University School of Medicine, Morgantown, WV, United States; ^6^ Department of Pathology and Critical Care Medicine, Johns Hopkins School of Medicine, Baltimore, MD, United States; ^7^ Department of Anesthesiology and Critical Care Medicine, Johns Hopkins School of Medicine, Baltimore, MD, United States

**Keywords:** non-coding RNAs, microRNA, LncRNA–long noncoding RNA, mitochondria, intracellular communication, human health

Once considered solely as the powerhouse of eukaryotic cells, the mitochondrion is now known to be a hub for several other important cellular processes such as apoptosis, calcium homeostasis, regulation of innate immunity, amino acid metabolism, stem cell regulation and the cellular regulome, among others (Murphy and Steenbergen, 2011). Some of these functions are accounted for by the minimal circular 16,569-bp genome of human mitochondria (mtDNA), which contains the genes for 2 rRNAs, 22 tRNAs and 13 polypeptides (Chen et al., 2009). However, most mitochondrial proteins are nuclear-encoded and imported from the cytosol (Neupert and Herrmann, 2007). Lately, there is increasing evidence of bidirectional communication between mitochondria and the nucleus, coordinating multiple cellular functions (Soledad et al., 2019). This connection, nonetheless, is not limited to mitochondrial import of nuclear-encoded proteins and export of metabolites, but has also been shown to involve noncoding RNAs (ncRNAs) connecting both cellular compartments (Baradan et al., 2017; Macgregor-Das and Das, 2018; Soledad et al., 2019). Long noncoding RNAs (lncRNAs), as well as microRNAs (miRNAs) have been found inside mitochondria, both originating from the mtDNA (Mach et al., 2016; Fitzpatrick et al., 2019) and from the nucleus (Bandiera et al., 2011; Barrey et al., 2011). These mitochondria-associated ncRNAs have been shown to participate in intracellular communication, not only with the nucleus, but also with other cellular compartments (Srinivasan and Das, 2015; Baradan et al., 2017). Elucidation of the communication between mitochondria and the rest of the cell will constitute a very important piece in the grand puzzle of cellular homeostasis and pathogenesis.

Since the identification of miRNAs in the mitochondrial compartment in early 2011 (Barrey et al., 2011; Das et al., 2012), a myriad of publications on this Research Topic were produced in many cellular models and several mammalian species including human, mouse, rat and horse. Most of these reports provide novel information about how these ncRNAs within mitochondria modulate cellular function. Recent advancements in RNA-sequencing technology have enticed new researchers to begin investigating mitochondrial ncRNAs. Therefore, there is a great need for basic information and standardized guidelines, such as normalization controls and mitochondrial isolation procedures, to conduct rigorous research in this field. Here, we have gathered a great Research Topic of studies and review articles for the broader community, including areas that require further research and validation.

For this Research Topic, Liu and Shan presented a comprehensive review on mtDNA-encoded ncRNAs where they discussed the roles that ncRNAs play in cellular pathophysiology and the bi-directional communication between mitochondria and the nucleus. In another review, Giordani et al. have focused on small ncRNAs (mainly microRNAs) that are present in the mitochondrial compartment. These microRNAs are called “mitomiRs.” The authors discussed the emerging roles of mitomiRs in the bi-directional communication between mitochondria and the nucleus. In this article, the authors mainly focused on the role of mitomiRs in cellular metabolism, dynamics, and bioenergetics and how this could lead to the production of considerable amounts of reactive oxygen species (ROS) and increased mitochondrial permeability, which are among the hallmarks of cellular senescence. Huang et al. authored another review article where they discussed the cross-talk between mitochondria and other cellular organelles using ncRNAs as mediators. These authors summarized the implications of mitochondria-centric ncRNA trafficking with respects to cellular function.

This unique Research Topic of articles focusing on mitochondria-associated ncRNAs also includes original research articles which show the therapeutic potential of RNA transcripts that alter mitochondrial function as a means to provide protection against disease. For example, Rodrigues et al., demonstrate that intramuscular delivery of miR-1 reduces insulin resistance during obesity. It has been previously shown that miR-1 can translocate into the mitochondrion and alter mitochondrial gene expression. Furthermore, depending on the level of stress in skeletal myocytes, miR-1 can translocate into the mitochondrion from the cytoplasm and target mitochondrial mRNA (Zhang et al., 2014). These data could provide insight into the therapeutic potential of ncRNAs for obesity-induced skeletal muscle dysfunction (Rodrigues et al.). In another original study, Duroux-Richard et al. show that mitomiR therapeutics can be beneficial for macrophage differentiation, polarization, and function. The regulation of macrophage inflammatory pathways is governed specifically by mitomiRs and the implications in immune-mediated inflammatory disorders remain poorly understood. This study also suggests that this mitomiR-dependent control could be further enhanced through the transfer of mitochondria from donor to target cells, as a new strategy for mitomiR delivery. Mathuram et al. report that synthetic overexpression of a small ncRNA, mito-ncR-805, can increase Krebs cycle activity and mitochondrial OXPHOS, stabilize mitochondrial potential, accelerate cell division, and reduce the levels of the pro-apoptotic pseudokinase TRIB3. Previously, the same group identified mito-ncR-805 as being conserved in mammalian mitochondrial genomes, however there are shorter versions of mouse and human transcripts (mmu-CR805 and hsa-LDL1, respectively). Mito-ncR-805 acts as a retrograde signal between mitochondria to the nucleus (Blumental-Perry et al., 2020). In the present study, Mathuram et al. altered a functional sequence of the D-loop transcript, mito-ncR-805, and demonstrated that this could have a significant effect on cellular function. Nandi et al., using a rat model, both *in vitro* (H9c2 cell line) and *in vivo*, demonstrated that enhanced sympatho-excitation to the heart elicits cardiac miR-18a-5p/HIF-1α signaling that leads to mitochondrial abnormalities and consequential pathological cardiac remodeling. The goal of this study was to find a therapeutic option against hypertension-induced heart failure, as the hallmark of hypertensive heart disease are sympatho-excitation and cardiac mitochondrial abnormality. In this study, the authors identified the mechanism whereby miR-18a-5p can regulate ROS-induced mitochondrial transition pore opening via activation of pro-hypertrophy/fibrosis/inflammatory factors that induce pathological cardiac hypertrophy and fibrosis commonly observed in neurogenic hypertension (Nandi et al.).

Another interesting subject covered in this Research Topic is mitochondria-derived tRNA fragments (tRFs). This family of molecules was observed many years ago and were first thought to be degradation products of transcripts. However, with the advancement in sequencing technologies and meta-analysis of data, several groups have confirmed both the existence of tRFs in the cytoplasm and mitochondria, and more importantly, that they carry out biological functions. The review paper by Shaukat et al. provides general information on the types of tRF and their biological functions, with special focus on mitochondrial tRNA-derived fragments (mt-tRFs). The recent data on biogenesis of mt-tRFs and their putative functions are discussed in detail, taking into account the possible role of mt-tRNA modification patterns. Correlation of some mt-tRFs with specific human disorders raises their potential as biomarkers for several types of cancer, some infections and neurodegenerative diseases caused by mtDNA mutations. To further validate the importance of point mutations on mt-tRFs, Meseguer and Rubio published an original research article, where they demonstrated that MELAS (mitochondrial encephalomyopathy, lactic acidosis, and stroke-like episodes) disease, caused by the m.3243A>G mutation in the mitochondrial tRNALeu (UUR) gene, is associated with changes in the expression levels of several mt-tRFs. Importantly, mt-tRF Leu (UUR) mimic partially restored mitochondrial respiration in MELAS cybrid cells, which indicates the therapeutic and diagnostic potential of mt-tRFs. The authors also identified that Dicer and Ago2, two essential proteins in miRNA maturation and function, play an important role in mt-tRF biogenesis.

Finally, this Research Topic also incorporates a study related to the current ongoing SARS-CoV-2 pandemic. Pozzi performed an *in silico* analysis (Pozzi) and identified potential mitochondrial ncRNAs that could play an important role in the pathogenesis of SARS-CoV-2 replication and propagation, which could potentially underlie the basis for long COVID syndrome. More research is needed to better understand whether mitochondrial ncRNA(s) may lead to potential therapeutics for SARS-CoV-2 infection but this study serves as a basis for understanding the pathological mechanisms of SARS-CoV-2.

**FIGURE 1:**
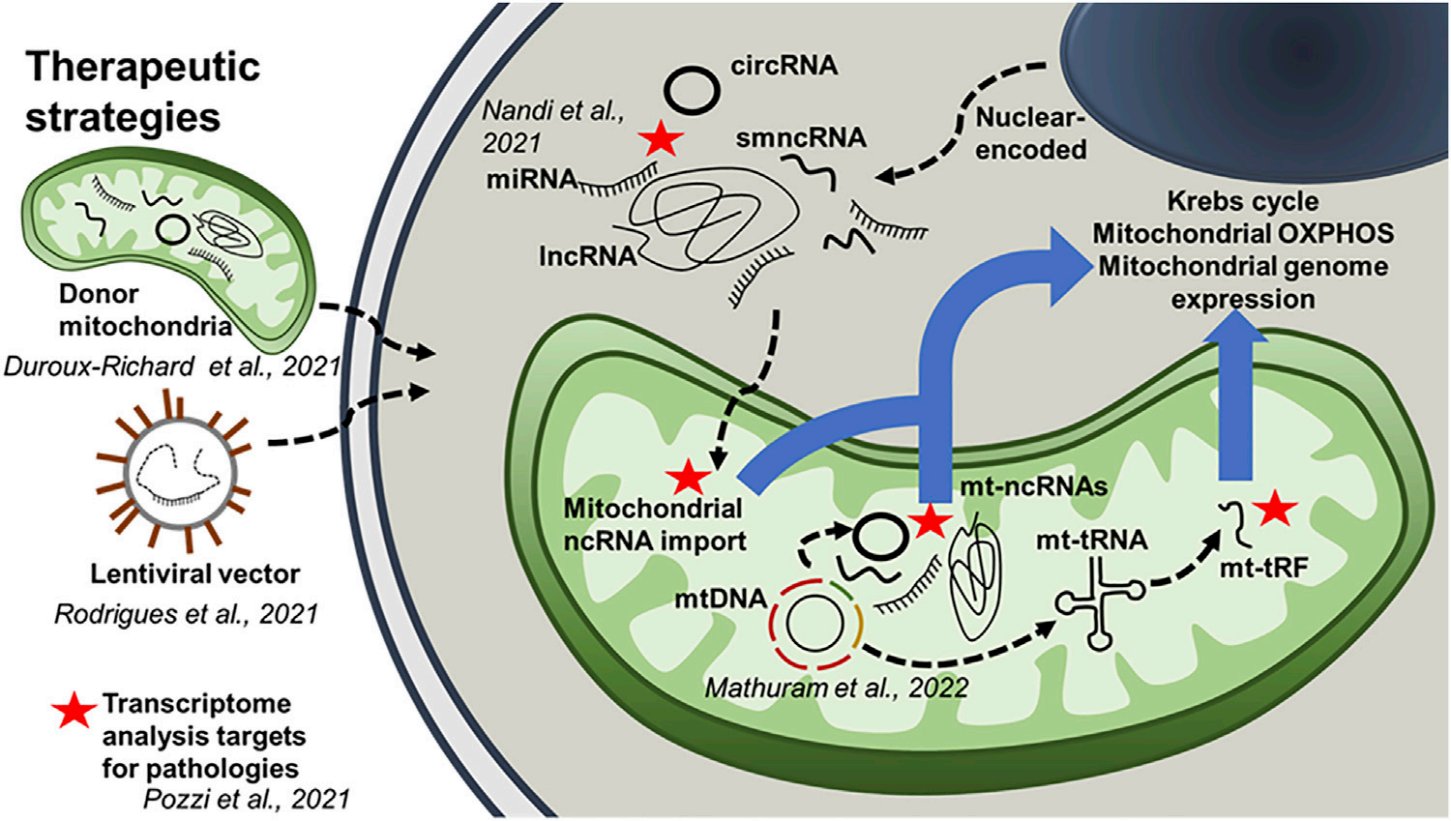
The role of mitochondria-associated ncRNAs. This schematic diagram integrates results obtained from the Research Topic of articles in this special edition. It highlights the potential role of mitochondria-associated ncRNAs in intracellular communication. The diagram also outlines several therapeutic strategies using ncRNAs.

We hope the reader finds this editorial and the articles it references to be useful in understanding the current state of the field regarding mitochondria-associated ncRNAs and their roles in intracellular communication, undoubtedly mediated by a myriad of long and small ncRNAs (Figure 1). Further studies into these processes will shed light on which pathways are involved in the mechanisms underlying normal and pathological involvement of ncRNAs in the communication between mitochondria and other organelles, particularly the nucleus. Ultimately, the expansion of our knowledge on these cellular events will lay the groundwork for the development of potential therapeutic strategies for different human pathologies.
